# Intraosseous pleomorphic adenoma of the mandible: A case report and literature review

**DOI:** 10.1097/MD.0000000000050405

**Published:** 2026-09-04

**Authors:** Lijuan Ma, Yao Shi, Ziyu Sun, Yonghai Song

**Affiliations:** a Department of Oral and Maxillofacial Surgery, Zibo Central Hospital, Zibo, Shandong, China.

**Keywords:** differential diagnosis, intraosseous pleomorphic adenoma, mandible, treatment

## Abstract

**Rationale:**

Salivary gland tumors account for 3% to 6% of all head and neck tumors, with pleomorphic adenoma (PA) being the most common benign type. However, primary intraosseous PAs arising from ectopic salivary tissue within the jawbones are exceptionally rare, with only a handful of cases reported in the literature. Clinically and radiographically, these lesions often mimic odontogenic pathologies, posing significant diagnostic challenges. This rarity and diagnostic ambiguity underscore the need to document and analyze such cases to improve clinical recognition and differential diagnosis.

**Patient concerns:**

We present a case of a patient with a mandibular lesion that clinically and radiographically mimicked a common lateral periodontal cyst, raising initial suspicion of an odontogenic pathology rather than a salivary gland-derived tumor.

**Diagnoses:**

Following comprehensive clinical evaluation, radiographic imaging, and histopathological examination, the lesion was diagnosed as a primary intraosseous PA of the mandible. To contextualize this diagnosis, a literature review was conducted, synthesizing data from all 14 known cases (including the current one) in a comprehensive table.

**Interventions:**

We conducted comprehensive preoperative examinations for the patient and informed them of the surgical risks. After obtaining their informed consent, the patient underwent a left mandibular lesion removal under general anesthesia. Postoperatively, anti-infection treatment was administered.

**Outcomes:**

This case report adds to the limited body of literature on primary intraosseous PAs, providing detailed clinical, radiographic, and histopathological findings that contribute to the understanding of this rare entity. The synthesized literature table serves as a valuable reference for clinicians managing similar jaw lesions.

**Lessons:**

Our report emphasizes the critical importance of including primary intraosseous PA in the differential diagnosis of jaw radiolucencies, especially in cases presenting with cortical perforation. Clinicians should remain vigilant to the possibility of rare salivary gland tumors even when lesions exhibit features typical of more common odontogenic pathologies to avoid misdiagnosis and ensure appropriate management.

## 1. Introduction

Salivary gland neoplasms account for roughly 3% to 6% of all head and neck tumors.^[1]^ Pleomorphic adenoma (PA) is the most common salivary gland neoplasm, with the majority occurring in the parotid gland.^[2]^ Although benign intraosseous salivary gland neoplasms of the jaws are exceedingly rare, their diagnosis requires strict evaluation to confirm a central bony origin.^[3]^ Clinically and radiographically, these lesions can mimic odontogenic cysts or tumors; as a result, their diagnosis tends to be unexpected.^[4]^ They may present as asymptomatic, well-circumscribed masses discovered incidentally during dental scans, or they can cause symptoms such as painless swelling. This report describes a case of intraosseous PA and reviews the relevant literature.

## 2. Case presentation

In January 2023, a 66-year-old Asian male presented with a 2-month history of swelling in the left posterior mandible and numbness in his left lower lip. His chief complaints were pain and swelling in the area, which interfered with eating. Six months prior, his left mandibular second molar had been extracted due to loosening. His medical history was significant for type 2 diabetes, diagnosed 5 years earlier.

**Figure 1.**
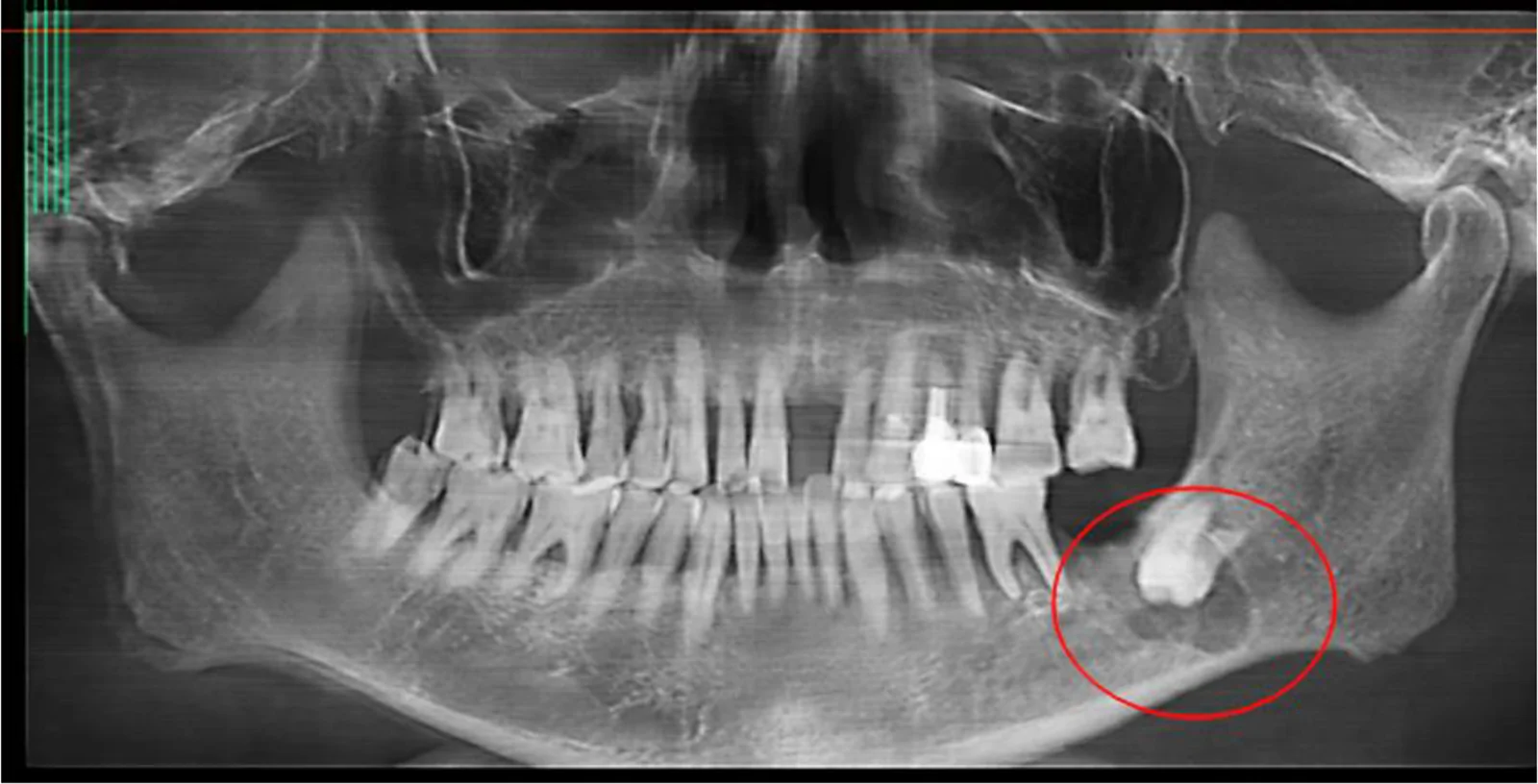
CBCT scan showing a well-circumscribed, unilocular radiolucency with defined borders, enveloping part of tooth 38. Tooth 37 was previously extracted. CBCT = cone-beam computed tomography.

**Figure 2.**
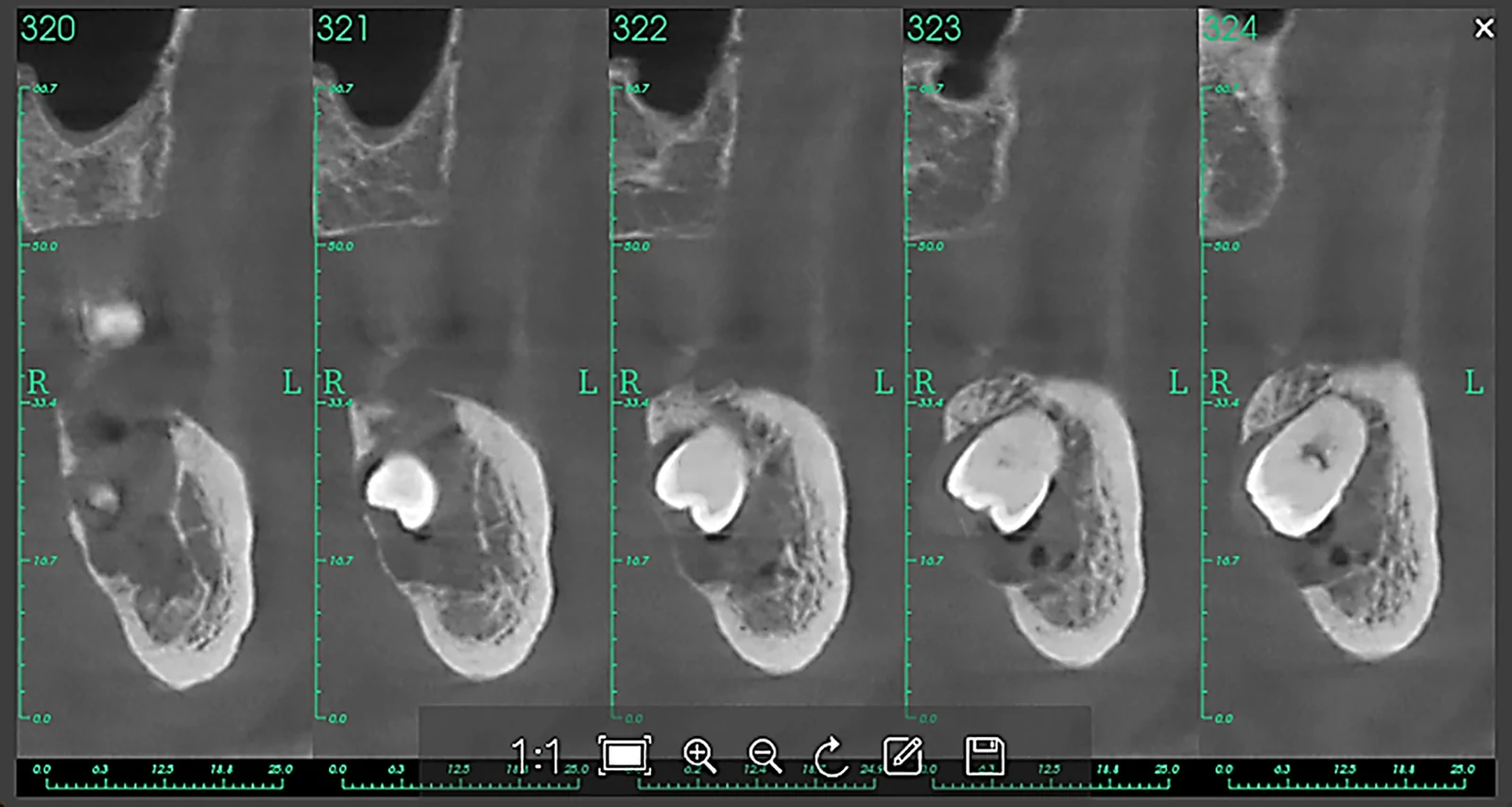
CBCT scan showing perforation of the lingual cortical plate and invasion of the left mandibular neurovascular bundle by the lesion. CBCT = cone-beam computed tomography.

Clinical examination revealed facial asymmetry due to soft tissue swelling on the left side. His maximum mouth opening was limited to 2.5 cm. The mucosa overlying the anterior edge of the left mandibular ramus was reddish but normal in texture. No cervical lymphadenopathy was detected. Cone-beam computed tomography revealed a well-circumscribed, unilocular radiolucency with defined borders (Fig. 1). The lesion enveloped part of the third molar (tooth 38) and had perforated the lingual cortical plate, invading the left mandibular neurovascular bundle (Fig. 2). Based on the clinical and radiographic findings, the differential diagnosis included a lateral periodontal cyst, odontogenic keratocyst, periradicular cyst, or a metastatic lesion.

Surgery was performed in March 2023. During surgery, after reflecting the gingival tissues, the lesion was found to have destroyed the lingual cortical plate. The mass was unusually firm to probing, did not resemble granulation tissue, and was nonhemorrhagic. It was readily accessible and removed with curettes. The mandibular neurovascular bundle was visible at the base of the surgical defect. The bony cavity was smoothed with a surgical bur to remove any residual tissue, and the adjacent first and third molars (teeth 36 and 38) were extracted. The excised specimen was a tan and brown mass measuring 1.5 × 1.0 × 1.0 cm. The surgical site healed uneventfully within 2 weeks, though the numbness in the left lower lip persisted. To date, the patient has undergone 5 examinations and has not shown any sign of recurrence.

## 3. Histopathologic examination

**Figure 3.**
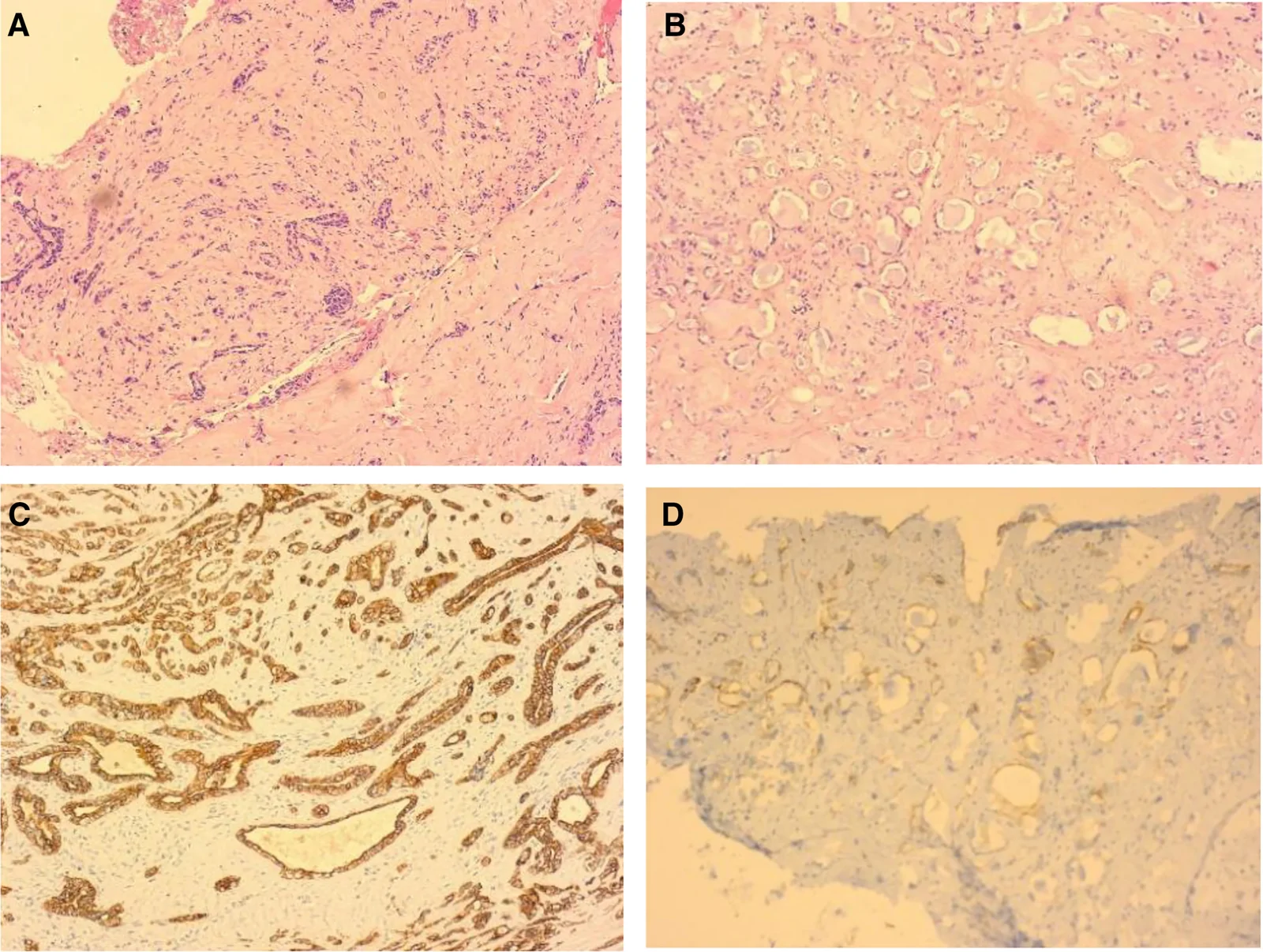
Histopathology of the pleomorphic adenoma. (A, B) Hematoxylin and eosin staining (100× magnification) reveals (A) neoplastic epithelial and myoepithelial cells in cord-like and glandular patterns and (B) myxoid and chondroid areas with calcification. (C, D) Immunohistochemical stains show strong reactivity of neoplastic cells for myoepithelial markers 34βE12 and CK8/18.

Microscopic examination revealed a well-circumscribed, benign neoplastic proliferation of salivary gland tissue within a fibrous stroma showing hyaline degeneration and focal calcification. The lesion contained cords, nests, and glandular tubules of abnormally proliferating cells. It also featured multiple myxoid and chondroid (cartilage-like) areas, along with scattered, irregular foci of metaplastic calcification and mineralization (Fig. 3A and B).

Immunohistochemical staining showed that the lesional cells were strongly reactive for the keratins 34βE12 and CK8/18 (Fig. 3C and D). The lesional cells also demonstrated positivity for Vimentin, epithelial membrane antigen, and S-100. Vimentin expression was specifically localized to endothelial cells, fibroblasts, and a minority of the neoplastic cells. Both positive and negative controls stained appropriately. These histopathological features confirmed the diagnosis of PA.

## 4. Discussion

PAs overwhelmingly arise from salivary gland tissue and are the most common tumor type affecting these organs.^[5]^ They occur in both major and minor salivary glands. The occurrence of PA in other locations – such as the middle ear, mastoid, nose, and cervical lymph nodes – is extremely rare,^[6–9]^ as are sporadic reports in the extremities, trunk, and genitals.^[10]^ For conventional PAs, women are affected more commonly than men (an approximate 2:1 ratio), with a peak incidence between the third and sixth decades of life, though cases have been reported in all age groups.^[2]^

Typically, PA presents as a painless, insidious, and slowly progressing tumor that can reach a large size if left untreated. Immunohistochemical staining for PA shows reactivity for cytokeratin, which is most intense in the duct-like epithelial cells. Smooth muscle actin and vimentin reactivity are seen in spindle-shaped cells, while glial fibrillary acidic protein positivity is more consistently observed in myxoid areas. S-100 staining is variable but may be intense and present in all cell types.

Reports of PAs originating centrally within the jaws are exceedingly rare. A comprehensive literature review identified 13 such cases; our report brings the total to 14 (Table 1).^[3,4,10–15]^ In this small cohort, men and women were affected equally, with patient ages ranging from 11 to 80 years. The oldest patient, an 80-year-old, was identified in a literature review by Brookstone and Huvos.^[3]^ The most common clinical presentation was swelling, followed by pain and interference with dentures. Consistent with our findings, most reported cases involved expansion of the affected jawbone. The lesion in our patient also caused erosion of the cortical plate. The imaging findings for this lesion – a radiolucency involving tooth 38 with unclear boundaries near the neurovascular bundle – resemble those of cystic lesions of the jaw, creating potential for misdiagnosis.

**Table 1:** Summary of previously published cases of intraosseous pleomorphic adenoma.

Reference	Number of cases	Patient demographics: age (yr), sex (M/F)	Site	Clinical presentation
Brookstone and Huvos^[3]^	5*	32–80 (2M, 2F, 1NA)	3 mandible	Swelling and pain
			1 maxilla	
			1 NA	
Aver-De-Araujo et al^[4]^	1	31 (F)	Maxilla	Swelling and inability to wear dentures
Ojha et al^[10]^	1	65 (F)	Mandible	Swelling and interference with wearing dentures
Aghaghazvini and Aghaghazvini^[11]^	1	38 (F)	Mandible	Mild pain and paresthesia of the mental region
Ferretti et al^[12]^	2	19 (F)	Both maxilla	Swelling and drainage
		22 (M)		
Bajpai^[13]^	1	11 (M)	Mandible	Painless swelling
Manola et al^[14]^	1	56 (M)	Maxilla	Asymptomatic
Manerikar et al^[15]^	1	34 (M)	Mandible	Swelling and pain
Current case	1	66 (M)	Mandible	Swelling and numbness of lower lip

NA = not available.

* Data are from 5 cases of benign mixed tumor identified in the literature review portion of their study, not from the primary cohort.

Several theories have been proposed to explain the origin of intraosseous salivary gland neoplasms. Most suggest neoplastic transformation of ectopic salivary gland tissue derived from embryonically entrapped rests. Additionally, Johnston et al hypothesized that some intraosseous neoplasms may originate from the direct invasion of a soft tissue salivary gland tumor into the jawbone.^[16]^

A diagnosis of a primary intraosseous salivary gland tumor requires that 3 criteria be met: no concurrent or preexisting primary tumor in the major or minor salivary glands; radiographic evidence of osteolysis with intact cortical plates; and a definitive histological diagnosis of a salivary gland neoplasm.^[17–19]^ A Stafne defect, which is a lingual concavity of the mandible containing salivary gland tissue, is not considered a true intraosseous lesion because the tissue is not fully encased by bone.^[4]^ Reports of central salivary gland tumors are considered most reliable when the mandible is the site of origin.

While intraosseous PA occurs more frequently in the mandible than in the maxilla, the mandible lies in close proximity to the submandibular and sublingual glands. Salivary gland tumors in the maxilla may originate from the submucosal secretory glands within the maxillary sinus and invade the sinus through minor defects in the cortical bone, and have a lower incidence than those in the mandible.^[14]^ In our case, there was no evidence of a primary tumor elsewhere in the head and neck. However, the case did not meet the criterion requiring cortical bone integrity, as the lesion perforated the lingual plate. Despite this deviation, the lesion’s location and histopathology led to the final diagnosis of a primary intraosseous PA of the mandible.

Surgery is the preferred treatment for intraosseous PAs. Based on the patient’s clinical manifestations and imaging features, thorough curettage of the lesion within the jawbone should first be performed, followed by smoothing of the surgical cavity walls with a surgical bur to remove the diseased tissue as thoroughly as possible.^[15]^ Due to the limited number of cases reported in the literature, under all circumstances, these lesions need careful follow-ups.

## 5. Conclusion

Intraosseous PAs are rare lesions that pose a significant diagnostic challenge. Because they develop in tooth-bearing areas and are often closely associated with teeth, their clinical and radiographic features can mimic more common odontogenic cysts, which makes a preoperative diagnosis difficult. The literature on these tumors is sparse, and most case reports lack long-term follow-up data, which prevents a meaningful analysis of recurrence rates. Therefore, definitive treatment followed by long-term clinical and radiographic follow-up is advisable for all patients.

## Author contributions

**Conceptualization:** Lijuan Ma, Yonghai Song.

**Data curation:** Lijuan Ma.

**Formal analysis:** Lijuan Ma, Yao Shi.

**Investigation:** Lijuan Ma.

**Supervision:** Yao Shi.

**Validation:** Lijuan Ma, Yao Shi, Ziyu Sun, Yonghai Song.

**Writing – original draft:** Lijuan Ma.

**Writing – review & editing:** Lijuan Ma, Yao Shi, Ziyu Sun, Yonghai Song.
